# Illiteracy, low educational status, and cardiovascular mortality in India

**DOI:** 10.1186/1471-2458-11-567

**Published:** 2011-07-15

**Authors:** Mangesh S Pednekar, Rajeev Gupta, Prakash C Gupta

**Affiliations:** 1 Healis Sekhsaria Institute for Public Health, CBD Belapur, Navi Mumbai, Maharashtra-400614, India; 2 Department of Medicine, Fortis Escorts Hospital, Malviya Nagar, Jaipur-302017, India

## Abstract

**Background:**

Influence of education, a marker of SES, on cardiovascular disease (CVD) mortality has not been evaluated in low-income countries. To determine influence of education on CVD mortality a cohort study was performed in India.

**Methods:**

148,173 individuals aged ≥ 35 years were recruited in Mumbai during 1991-1997 and followed to ascertain vital status during 1997-2003. Subjects were divided according to educational status into one of the five groups: illiterate, primary school (≦ 5 years of formal education), middle school (6-8 years), secondary school (9-10 years) and college (> 10 years). Multivariate analyses using Cox proportional hazard model was performed and hazard ratios (HRs) and 95% confidence intervals (CIs) determined.

**Results:**

At average follow-up of 5.5 years (774,129 person-years) 13,261 deaths were observed. CVD was the major cause of death in all the five educational groups. Age adjusted all-cause mortality per 100,000 in illiterate to college going men respectively was 2154, 2149, 1793, 1543 and 1187 and CVD mortality was 471, 654, 618, 518 and 450; and in women all-cause mortality was 1444, 949, 896, 981 and 962 and CVD mortality was 429, 301, 267, 426 and 317 (p_trend _< 0.01). Compared with illiterate, age-adjusted HRs for CVD mortality in primary school to college going men were 1.36, 1.27, 1.01 and 0.88 (p_trend _< 0.05) and in women 0.69, 0.55, 1.04 and 0.74, respectively (p_trend _> 0.05).

**Conclusions:**

Inverse association of literacy status with all-cause mortality was observed in Indian men and women, while, for CVD mortality it was observed only in men.

## Background

Illiteracy and low educational status are highly prevalent in low income countries. It is well known that poverty is associated with greater ill health and mortality [1] and low educational status is a major determinant of disease as well as mortality [2]. Low educational status is associated with under-nutrition, greater infant and maternal mortality, and acute and chronic infections [1]. In high and middle income countries it is also associated with increased incidence and mortality from chronic diseases such as cardiovascular disease (CVD), chronic respiratory diseases and cancer [2,3].

In developing countries CVDs (coronary heart disease and stroke) are considered to be more prevalent in higher socioeconomic status (SES) and more literate subjects [4]. Using the corollary of developed North American and Western European countries where the diseases were more frequent among the more literate subjects till 1960's and then became more in the less literate [4], it has been argued that the burden of CVDs could be shifting and could be more in the poor subjects in countries in economic transition such as India [5]. However, reliable national SES- or literacy-specific mortality statistics do not exist here. Many cardiovascular risk factor epidemiological studies in mid and late 20^th ^century have reported that the risk factors are more in upper SES subjects as compared to the poor [6], although some studies reported that risk factors could be more in poor especially where the problem of illiteracy is high [7]. Recent case-control studies have reported that SES, as measured by educational status, is inversely related to acute myocardial infarction [8,9] and observational studies have reported that low SES subjects are more likely to die from acute coronary events as compared to the rich [10]. To determine association of educational status as marker of SES with cardiovascular mortality we performed a prospective cohort epidemiological study in Mumbai, India.

## Methods

### Recruitment

The Mumbai Cohort Study was conducted in the main city of Mumbai (India), with mortality as the endpoint. A total of 148,173 persons aged ≥ 35 years were recruited during 1991-1997. House-to-house interviews were conducted face-to-face using a structured questionnaire. Electoral rolls, organized by area with a polling station of 1,000-1,500 individuals as the smallest geographical unit, were used as the sampling frame. The electoral rolls provided name, age, sex, and address of all the individuals aged ≥ 18 years. We excluded polling stations that served upper-middle-class and upper-class housing complexes because of security issues (i.e., they were essentially ''gated communities''). For a selected polling station, all eligible people (aged ≥ 35 years) listed on its electoral roll were interviewed in local languages (Marathi, Hindi) by trained field supervisors by using handheld computers (electronic diaries) but the information was recorded in English. The study satisfies all the criteria regarding the ethical treatment of human subjects, especially those formulated by the Indian Council of Medical Research (ICMR). This study was approved by independent institute review board (Healis-IRB) formulated as per the guideline provided by ICMR (which confirmed to Helsinki declaration and to local legislation). Participatory oral consent was obtained from all participants at the time of recruitment. Details regarding the recruitment procedures and measurements have been published previously [11,12].

### Data sources

The baseline survey included the following components: 1) anthropometry to measure weight (using a bathroom scale that was calibrated to 100 gram amounts; staff recorded to the nearest kilogram) and height (using a specially constructed instrument consisting of a steel platform to which was attached a steel measuring tape that was calibrated to the nearest millimetre; staff recorded to the nearest cm); and 2) Interviewer administered structured questionnaire [11-14]. For the present study, data regarding age, sex, education (as proxy for SES), religion, mother tongue, height, weight, body mass index (BMI), and details on tobacco use were abstracted from the baseline data [11-14]. Subjects were classified according to their educational status into illiterate, primary school (≤ 5 years of formal education), middle school (6-8 years), secondary school (9-10 years) and college (> 10 years). Subjects were also broadly classified as having never used tobacco, or being a current or former user of smokeless tobacco only, or being a current or former smoker only or both (includes those who smoke and use smokeless tobacco).

### Follow-up

**Figure 1:**
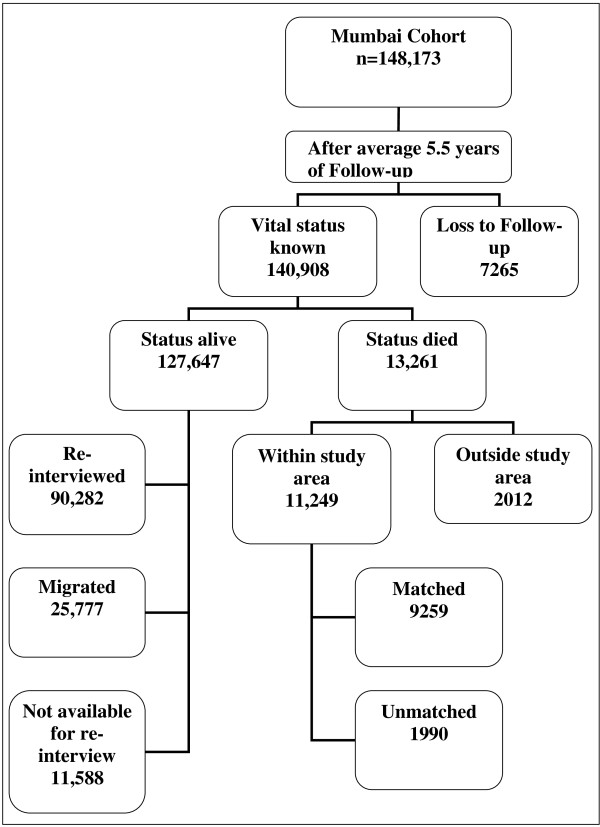
**Flow diagram of house-to-house follow-up, Mumbai Cohort Study**.

An active house-to-house follow-up was conducted on average 5.5 years after the baseline survey. The field supervisors were provided with the list of names and addresses of cohort members and were instructed to revisit each person. If the person was alive and available, a face-to-face re-interview was conducted. If the person was reported to have died, the date and place of death were recorded with extra questioning and care. Permanent migration, while the subject was alive, from the study area was considered as withdrawal from the study, and the date of migration was noted. The re-interviews were conducted during 1997-2003. The results of follow-up are shown in Figure 1 and additional file 1 as reported earlier [11-14].

### Cause of death

The deaths recorded during the follow-up were linked with the dataset obtained from the municipal corporation death registers. In Mumbai, almost all the deaths are registered and medically certified. For matched deaths, the underlying cause of death was derived from the cause information copied from the corporation death registers and then coded according to the ICD-10 guidelines. Cause specific analyses were performed for various circulatory system related deaths (ICD-10 codes I00-99, will be referred as CVD here after) such as ischemic heart diseases (I20-25, referred as IHD) and cerebrovascular diseases (I60-69, referred as stroke). For 1685 randomly selected matched deaths, an independent field check was performed and matching was found to be nearly 100% accurate [11].

### Statistical analysis

Methodological details regarding anthropometric measurements, and information collected from the structured questionnaire have been published [11-14]. Follow-up methodology has been reported [11]. Causes of deaths are reported in percent. Age-adjusted rates for all-cause, CVD, IHD and stroke mortality were determined separately for men and women and reported as deaths per 100,000 subjects. Adjusted survival curves have been plotted for various educational groups for all-cause and CVDs. The association between various educational groups and all-cause, CVD, IHD, and stroke deaths are presented as hazard ratios (HRs) and 95% confidence intervals (CIs) derived from multivariate Cox proportional hazards regression modelling using SPSS 13.0. The response variable, death, was coded as a dichotomous variable, and the time to event or censoring was regarded as a continuous variable. Age, smoking or tobacco use and body mass index (BMI) were added to the model as independent variables using stepwise regression analyses. Adjusted HRs and 95% CIs were estimated separately for men and women. A population attributable fraction (PAF) [11] was calculated using a formula ∑pd_i_(RR_i_-1)/RR_i_, where 'pd_i_' represents the proportion of the total deaths in the population arising from the i^th ^exposure category and RR_i _is the (adjusted) RR for the i^th ^exposure category (relative to the reference or unexposed stratum).

## Results

Baseline characteristics of the study subjects are shown in Table 1. There were 88,658 men and 59,515 women in the cohort. Most of the subjects were in age-groups 45-59 years. Illiteracy was more among women (45.3%) than men (17.0%). Only 15.8% men and 5.9% women had more than secondary level education. High prevalence of overweight or obesity (BMI ≥ 25 kg/m^2^) was also observed in both men (20.2%) and women (29.4%). Prevalence of any tobacco use was also high (men 69.9% and women 59.7%). Around 80% subjects were Hindu while over 60% reported Marathi as their mother tongue.

**Table 1:** Demographic details of the study subjects by educational status*for men, Mumbai Cohort Study, Mumbai, Maharashtra, India

Men	Illiterate (n = 15091)	Primary school (n = 33549)	Middle school (n = 26075)	Secondary school (n = 8308)	College (n = 5635)	Total (N = 88658)
**Age groups**						
35 to 39	3.9%	7.5%	13.2%	13.6%	12.1%	9.4%
40 to 44	3.9%	7.0%	10.2%	11.3%	9.1%	7.9%
45 to 49	24.4%	23.1%	30.3%	33.1%	29.0%	26.7%
50 to 54	18.6%	15.6%	16.1%	15.1%	14.3%	16.1%
55 to 59	13.8%	13.1%	10.9%	10.0%	11.3%	12.2%
60 to 64	14.6%	12.6%	8.8%	6.7%	9.3%	11.1%
65 to 69	9.2%	9.5%	5.3%	4.9%	6.5%	7.6%
70 & up	11.5%	11.6%	5.3%	5.3%	8.3%	8.9%

**Religion**						
Hindu	68.5%	77.1%	79.1%	81.9%	79.6%	76.8%
Muslim	25.7%	15.4%	14.1%	8.4%	14.1%	16.0%
Buddhist	4.7%	4.8%	3.6%	4.3%	1.8%	4.2%
Christian	1.0%	2.4%	2.8%	4.1%	3.4%	2.5%
Others	0.2%	0.3%	0.3%	1.3%	1.1%	0.4%

**Mother tongue**						
Marathi	37.1%	57.9%	59.1%	69.5%	53.4%	55.5%
Hindi	34.2%	13.8%	15.2%	8.1%	9.8%	16.9%
Gujarati	4.2%	10.2%	11.1%	6.2%	19.1%	9.6%
Urdu	13.1%	9.4%	6.6%	6.9%	7.6%	8.9%
South Indian	11.3%	8.5%	7.8%	8.9%	9.9%	8.9%
Others	0.2%	0.1%	0.2%	0.5%	0.1%	0.2%

BMI**(kg/m^2^)						
Normal	64.1%	62.3%	61.7%	63.3%	60.3%	62.4%
Thin	12.9%	10.1%	7.7%	6.0%	4.7%	9.2%
Very Thin	5.1%	4.6%	3.5%	2.5%	1.5%	3.9%
Extremely Thin	5.5%	5.1%	3.7%	2.8%	1.6%	4.3%
Overweight	10.9%	15.7%	20.2%	22.4%	26.7%	17.5%
Obese	1.5%	2.3%	3.2%	3.0%	5.2%	2.7%

**Tobacco usage**						
Never-user	21.6%	23.8%	35.5%	37.3%	55.2%	30.1%
Smokeless	39.9%	42.8%	37.0%	35.3%	20.3%	38.5%
Smoker	14.8%	18.6%	14.0%	17.5%	17.1%	16.4%
Both***	23.8%	14.8%	13.5%	10.0%	7.5%	15.0%

**Women**	**Illiterate** **(n = 26959)**	**Primary school** **(n = 20850)**	**Middle school** **(n = 8196)**	**Secondary school** **(n = 2536)**	**College** **(n = 974)**	**Total** **(N = 59515)**

**Age groups**						
35 to 39	14.6%	27.4%	42.7%	47.5%	44.4%	24.8%
40 to 44	12.9%	19.2%	22.5%	20.0%	22.4%	16.9%
45 to 49	14.9%	16.9%	14.4%	11.4%	11.3%	15.4%
50 to 54	15.6%	12.8%	9.5%	7.8%	8.1%	13.3%
55 to 59	12.5%	9.0%	5.6%	5.3%	5.1%	9.9%
60 to 64	13.1%	7.3%	3.1%	4.6%	4.5%	9.2%
65 to 69	7.4%	4.0%	1.4%	2.1%	2.4%	5.1%
70 & up	8.9%	3.5%	0.9%	1.3%	1.8%	5.5%

**Religion**						
Hindu	79.2%	83.8%	85.6%	84.1%	85.9%	82.0%
Muslim	8.0%	5.6%	5.9%	4.1%	3.6%	6.7%
Buddhist	11.4%	5.2%	3.7%	3.0%	1.1%	7.6%
Christian	1.2%	4.7%	4.3%	7.1%	5.5%	3.1%
Others	0.2%	0.7%	0.5%	1.7%	3.8%	0.6%

**Mother tongue**						
Marathi	74.2%	75.6%	78.9%	74.7%	70.6%	75.3%
Hindi	10.0%	4.0%	6.1%	1.8%	4.9%	6.9%
Gujarati	4.7%	6.9%	4.1%	6.0%	8.2%	5.5%
Urdu	4.0%	4.0%	2.4%	3.4%	2.7%	3.8%
South Indian	6.7%	9.1%	7.7%	12.6%	13.0%	8.0%
Others	0.4%	0.3%	0.9%	1.5%	0.5%	0.5%

BMI**(kg/m^2^)						
Normal	52.4%	50.5%	50.1%	52.3%	48.0%	51.4%
Thin	11.2%	7.6%	7.7%	5.8%	4.1%	9.1%
Very Thin	5.4%	3.4%	3.9%	3.3%	1.4%	4.3%
Extremely Thin	7.8%	4.3%	4.2%	2.5%	1.5%	5.7%
Overweight	18.0%	25.8%	25.9%	27.8%	34.4%	22.5%
Obese	5.1%	8.4%	8.2%	8.3%	10.5%	6.9%

**Tobacco usage**						
Never-user	25.4%	45.8%	58.6%	74.0%	89.3%	40.3%
Smokeless	73.8%	53.9%	41.3%	25.9%	10.5%	59.3%
Smoker	0.4%	0.2%	0.1%	0.1%	0.2%	0.3%
Both***	0.3%	0.1%	0.1%	0.0%		0.2%

*Illiterate, Primary school (≤ 5 years of formal education), Middle school (6-8 years), Secondary school (9-10 years) and College (> 10 years)

**BMI body mass index = weight (kg)/height(m)^2^; BMI (kg/m2) categories were defined as follows: extremely thin (< 16.0); very thin (16.0 to < 17.0); thin (17.0 to < 18.5); normal (18.5 to < 25.0); overweight (25.0 to < 30.0); and obese (≥ 30.0)

***includes those who smoke and use smokeless tobacco

During follow-up, of the total recruited subjects 7265 could not be traced; the most common reason was the demolition of their residential buildings (6452 subjects). No differences in baseline variables were observed in subjects whose data were available as compared to those lost to follow-up (additional file 1). Among the remaining 140,908 subjects, 13,261 (9.4%) persons died while 127,647 were alive (of which 25,777 subjects had migrated outside study area) at the end of follow-up period. Of the total 13,261 deaths, 11,249 died within study area and among those died within study area 9259 deaths (72.3%) were matched and coded using ICD-10 (Figure 1). Details regarding the matching and coding of underlying causes of deaths published elsewhere [11-14]. For 260 deaths date of expiry was found to precede the date of recruitment; hence these subjects were excluded. Detailed investigation of a sample of these deaths revealed that the deaths had occurred very close to the date of recruitment of these subjects. Thus only 13,001 deaths were available for final analysis.

The subjects were followed for a mean of 5.5 years and 774,129 person-years were observed. The major causes of deaths in different educational groups are shown in Table 2. CVDs were the largest proportion of cause of death in men and women across all the educational groups. Age adjusted all-cause mortality per 100,000 in men for different educational groups was 2154 in illiterate, 2149 in primary school, 1793 in middle school, 1543 in secondary school and 1187 in college and in women it was 1444, 949, 896, 981 and 962, respectively. In different educational groups CVD mortality was 471, 654, 618, 518 and 450 in men and 429, 301, 267, 426 and 317 in women; IHD mortality was 234, 371, 401, 349 and 338 in men and 180, 133, 131, 227 and 96 in women and stroke mortality was 124, 135, 107, 59 and 31 in men and 87, 67, 58, 47 and 50 in women.

**Table 2:** Major causes of death (% of total deaths) in various educational groups*, Mumbai Cohort Study, Mumbai, Maharashtra, India

Men	Illiterate	Primary school	Middle school	Secondary school	College
Number of deaths	1904	4507	2197	597	384
Major causes of death (column %)	Cardiovascular (23.5) Respiratory (10.8) Other medical (10.4) Tuberculosis (7.4) Cancer (4.2)	Cardiovascular (32.5) Other medical (11.0) Respiratory (9.1) Tuberculosis (7.5) Cancer (5.9)	Cardiovascular (33.5) Other medical (10.8) Respiratory (8.6) Tuberculosis (7.4) Cancer (6.1)	Cardiovascular (31.3) Other medical (12.9) Tuberculosis (8.4) Cancer (7.0) Respiratory (6.7)	Cardiovascular (38.0) Other medical (9.4) Cancer (7.8) Tuberculosis (5.2) Respiratory (4.4)

**Women**	**Illiterate**	**Primary school**	**Middle school**	**Secondary school**	**College**

Number of deaths	2417	762	160	54	19
Major causes of death (column %)	Cardiovascular (29.8) Other medical (14.0) Respiratory (13.4) Tuberculosis (5.7) Cancer (4.5)	Cardiovascular (30.7) Other medical (15.1) Tuberculosis (10.6) Respiratory (7.6) Cancer (6.0)	Cardiovascular (24.4) Other medical (14.4) Tuberculosis (11.3) Respiratory (10.0) Cancer (9.4)	Cardiovascular (44.4) Other medical (18.5) Tuberculosis (9.3) Respiratory (5.6) Cancer (3.7)	Cardiovascular (36.8) Tuberculosis (15.8) Cancer (10.5) Respiratory (5.3) Other medical (5.3)

*Illiterate, Primary school (≤ 5 years of formal education), Middle school (6-8 years), Secondary school (9-10 years) and College (> 10 years)

**Figure 2:**
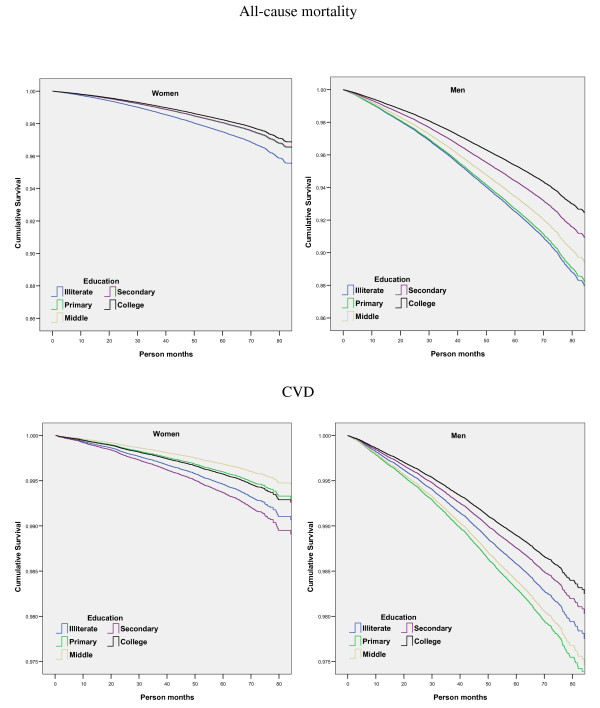
**Multivariate adjusted survival curves for all-cause and CVD mortality, Mumbai Cohort Study**.

Adjusted survival curves for all-cause and CVD mortality in men and women for different educational groups are shown in Figure 2. In men the greatest mortality was observed in illiterate and primary school men with better survival in more literate groups while in women no such clear associations was observed. Crude and adjusted HRs and 95% CIs for all-cause, CVD, IHD and stroke mortality are shown in Tables 3 (men) and 4 (women). All-cause mortality was highest in illiterate men and women and was used as a reference category for estimating HRs throughout the analysis. Compared to illiterate, the age-adjusted HRs were lower in other groups in men (1.00, 0.84, 0.71 and 0.55) as well as in women (0.68, 0.61, 0.64 and 0.57) with a significant negative trends (p < 0.001). For CVD mortality age adjusted HRs were higher in primary as well middle school men than illiterates (Table 3); in contrast, it was lower in women (Table 4). Most literate (> 10 years of formal education, i.e. college) men and women had the lowest CVD mortality (Table 3, 4). Multivariate adjustment for other available confounders such as various forms of tobacco use, BMI, religion and mother tongue attenuated HRs but did not nullify the association for all-cause as well as CVD mortality in men and women.

**Table 3:** Person years, number of deaths, hazard ratios (HRs) and 95% confidence intervals (CIs) for all-cause, CVD, IHD and stroke mortality in men stratified by educational groups*, Mumbai Cohort Study, Mumbai, Maharashtra, India

Men	Illiterate	Primary school	Middle school	Secondary school	College
**Person years**	69,064	165,757	135,193	44,003	29,876

**All-deaths (n)**	1904	4507	2197	597	384
**Death rates per 100000^2^**	2154	2149	1793	1543	1187
HR^1^ 95% CI	1.0	0.98 (0.93-1.03)	0.58 (0.55-0.62)	0.48 (0.44-0.53)	0.46 (0.41-0.51)
HR^2^ 95% CI	1.0	1.00 (0.95-1.06)	0.84 (0.79-0.89)	0.71 (0.65-0.78)	0.55 (0.50-0.62)
HR^3^ 95% CI	1.0	1.02 (0.97-1.08)	0.89 (0.83-0.94)	0.75 (0.68-0.82)	0.60 (0.54-0.67)
HR^4^ 95% CI	1.0	0.98 (0.92-1.03)	0.87 (0.82-0.93)	0.74 (0.67-0.82)	0.61 (0.55-0.69)

**CVD^5 ^deaths (n)**	448	1465	737	187	146
**Death rates per 100000^2^**	471	654	618	518	450
HR^1^ 95% CI	1.0	1.33 (1.20-1.48)	0.80 (0.71-0.90)	0.62 (0.52-0.73)	0.71 (0.59-0.85)
HR^2^ 95% CI	1.0	1.36 (1.22-1.51)	1.27 (1.13-1.43)	1.01 (0.85-1.19)	0.88 (0.73-1.06)

HR^3^ 95% CI	1.0	1.37 (1.23-1.52)	1.30 (1.16-1.47)	1.02 (0.86-1.22)	0.90 (0.74-1.09)
HR^4^ 95% CI	1.0	1.20 (1.07-1.33)	1.13 (1.00-1.28)	0.88 (0.73-1.04)	0.78 (0.64-0.94)

**IHD^6 ^deaths (n)**	223	835	481	125	109
**Death rates per 100000^2^**	234	371	401	349	338
HR^1^ 95% CI	1.0	1.53 (1.32-1.78)	1.05 (0.89-1.23)	0.83 (0.66-1.03)	1.06 (0.84-1.33)
HR^2^ 95% CI	1.0	1.57 (1.36-1.82)	1.67 (1.42-1.96)	1.35 (1.08-1.68)	1.31 (1.04-1.64)
HR^3^ 95% CI	1.0	1.58 (1.37-1.84)	1.71 (1.45-2.00)	1.36 (1.09-1.70)	1.32 (1.04-1.66)
HR^4^ 95% CI	1.0	1.35 (1.16-1.57)	1.44 (1.22-1.70)	1.16 (0.93-1.46)	1.11 (0.87-1.40)

**Stroke^7 ^deaths (n)**	115	303	126	22	10
**Death rates per 100000^2^**	124	135	107	59	31
HR^1^ 95% CI	1.0	1.08 (0.87-1.34)	0.53 (0.41-0.68)	0.28 (0.18-0.45)	0.19 (0.10-0.36)
HR^2^ 95% CI	1.0	1.12 (0.90-1.39)	0.86 (0.67-1.11)	0.47 (0.30-0.74)	0.24 (0.12-0.45)
HR^3^ 95% CI	1.0	1.13 (0.91-1.40)	0.90 (0.70-1.16)	0.50 (0.32-0.79)	0.27 (0.14-0.51)
HR^4^ 95% CI	1.0	1.01 (0.81-1.25)	0.80 (0.62-1.04)	0.41 (0.26-0.66)	0.23 (0.12-0.44)

*Illiterate, Primary school (≤ 5 years of formal education), Middle school (6-8 years), Secondary school (9-10 years) and College (> 10 years)

^1 ^crude hazard ratios (HRs), ^2 ^adjusted for age, ^3 ^adjusted for age and tobacco use, ^4 ^adjusted for age, tobacco use, BMI, religion and mother tongue, ^5 ^all circulatory system related deaths (ICD-10 codes I00-99), ^6 ^Ischemic Heart Disease deaths (I20-25), ^7 ^cerebrovascular deaths (I60-69).

**Table 4:** Person years, number of deaths, hazard ratios (HRs) and 95% confidence intervals (CIs) for all-cause, CVD, IHD and stroke mortality in women stratified by educational groups*, Mumbai Cohort Study, Mumbai, Maharashtra, India

Women	Illiterate	Primary school	Middle school and above			
			Middle school	Secondary school	College	Sub total
**Person years**	147,971	116,063	46,739	14,145	5320	66,204

**All-deaths (n)**	2417	762	160	54	19	233
**Death rates per 100000^2^**	1444	949	896	981	962	918
HR^1^ 95% CI	1.0	0.41 (0.38-0.44)	0.21 (0.18-0.25)	0.24 (0.18-0.31)	0.22 (0.14-0.35)	0.22 (0.19-0.25)
HR^2^ 95% CI	1.0	0.68 (0.63-0.74)	0.61 (0.52-0.72)	0.64 (0.49-0.84)	0.57 (0.36-0.90)	0.61 (0.53-0.71)
HR^3^ 95% CI	1.0	0.72 (0.66-0.79)	0.66 (0.56-0.78)	0.72 (0.55-0.95)	0.66 (0.42-1.04)	0.67 (0.58-0.78)
HR^4^ 95% CI	1.0	0.78 (0.71-0.85)	0.70 (0.59-0.82)	0.77 (0.58-1.02)	0.70 (0.44-1.10)	0.71 (0.62-0.82)

**CVD^5 ^deaths (n)**	721	234	39	24	7	70
**Death rates per 100000^2^**	429	301	267	426	317	306
HR^1^ 95% CI	1.0	0.41 (0.35-0.47)	0.17 (0.12-0.23)	0.34 (0.23-0.51)	0.26 (0.13-0.56)	0.21 (0.16-0.27)
HR^2^ 95% CI	1.0	0.69 (0.60-0.80)	0.55 (0.40-0.76)	1.04 (0.69-1.57)	0.74 (0.35-1.56)	0.68 (0.53-0.87)
HR^3^ 95% CI	1.0	0.73 (0.62-0.85)	0.59 (0.42-0.82)	1.17 (0.77-1.77)	0.84 (0.40-1.79)	0.73 (0.57-0.95)
HR^4^ 95% CI	1.0	0.75 (0.64-0.88)	0.59 (0.42-0.82)	1.17 (0.77-1.79)	0.79 (0.37-1.69)	0.73 (0.56-0.94)

**IHD^6 ^deaths (n)**	303	106	18	12	3	33
**Death rates per 100000^2^**	180	133	131	227	96	142
HR^1^ 95% CI	1.0	0.44 (0.35-0.54)	0.18 (0.11-0.29)	0.40 (0.23-0.71)	0.27 (0.09-0.84)	0.24 (0.16-0.34)
HR^2^ 95% CI	1.0	0.73 (0.59-0.92)	0.59 (0.37-0.96)	1.20 (0.67-2.14)	0.74 (0.24-2.31)	0.74 (0.52-1.07)
HR^3^ 95% CI	1.0	0.78 (0.62-0.99)	0.65 (0.40-1.05)	1.39 (0.76-2.53)	0.87 (0.28-2.76)	0.82 (0.56-1.20)
HR^4^ 95% CI	1.0	0.78 (0.62-0.99)	0.61 (0.37-1.00)	1.36 (0.74-2.48)	0.77 (0.24-2.44)	0.78 (0.53-1.14)

**Stroke^7 ^deaths (n)**	145	51	9	4	1	14
**Death rates per 100000^2^**	87	67	58	47	50	54
HR^1^ 95% CI	1.0	0.43 (0.32-0.60)	0.19 (0.10-0.37)	0.28 (0.10-0.75)	0.19 (0.03-1.33)	0.21 (0.12-0.36)
HR^2^ 95% CI	1.0	0.72 (0.52-1.00)	0.60 (0.30-1.19)	0.85 (0.31-2.29)	0.51 (0.07-3.68)	0.65 (0.37-1.13)
HR^3^ 95% CI	1.0	0.76 (0.54-1.06)	0.64 (0.32-1.27)	0.94 (0.34-2.59)	0.58 (0.08-4.17)	0.69 (0.39-1.24)
HR^4^ 95% CI	1.0	0.78 (0.55-1.10)	0.64 (0.32-1.29)	0.95 (0.34-2.63)	0.57 (0.08-4.17)	0.70 (0.39-1.25)

*Illiterate, Primary school (≤ 5 years of formal education), Middle school (6-8 years), Secondary school (9-10 years) and College (> 10 years)

^1 ^crude hazard ratios (HRs), ^2 ^adjusted for age, ^3 ^adjusted for age and tobacco use, ^4 ^adjusted for age, tobacco use, BMI, religion and mother tongue, ^5 ^all circulatory system related deaths (ICD-10 codes I00-99), ^6 ^Ischemic Heart Disease deaths (I20-25), ^7 ^cerebrovascular deaths (I60-69)

## Discussion

This study shows that there is significant inverse association of literacy status with all-cause mortality in urban Indian men and women. In men the CVD mortality is also significantly greater in low educational status subjects while the association is not clear in women. The association of education and mortality (all-cause, CVD, IHD, and stroke) in both men and women appears to be influenced mainly by age, followed by tobacco usage and body mass index (surrogate for lipid and glucose metabolism abnormalities), religion, and mother tongue. The policy implication from this study could be improving the educational status may results in preventing ~9% premature male and female deaths in developing country populations such as in India.

Bertrand Russell almost a century ago highlighted the importance of education as catalyst of society's well being [15]. For the last 50 years, studies from developed countries have consistently reported that subjects with illiteracy and low educational status have greater all-cause, chronic disease as well as cardiovascular mortality [16-21]. Studies from developed countries have also reported that greater literacy is associated with better uptake of preventive lifestyles, lower prevalence of risk factors, early diagnosis and management of chronic disease risk factors, better quality of acute disease treatment, and better long-term treatment and compliance [22,23]. All these lead to lower incidence of CVD and lower short- and long-term mortality. Studies from developing countries are not clear on association of cardiovascular mortality or risk factors [5,7,24-28]. The present study shows that the more literate men had lower mortality from CVD. Greater CVD mortality among the less educated subjects could also be due to poor quality management and control of risk factors and, indeed, we have reported that status of hypertension awareness among this cohort is dismal (less than 10% awareness) indicating poor health literacy, poor control of risk factors and possibly greater event rates and mortality [29].

This study has multiple limitations and strengths. We obtained cause of death information from local death registries. Cause-of-death registries are often imprecise in India and this could be important in our study. On the other hand, the Mumbai registry is one of the oldest and most efficient systems of mortality ascertainment and thus the data are the best from this country [11]. We also validated the ascertainment of the causes of death in a random sub-sample with physician-defined cause and the results were consistent. Secondly, pre-existing diseases and drug therapy can substantially influence mortality from communicable as well as non-communicable diseases such as CVD and we have no data on them. One way to exclude significant pre-existing morbid conditions is to analyse data after exclusion of deaths in the first two years, but we were not able to perform such analyses due to fewer number of deaths were observed in more literate groups. Moreover, such analyses are more relevant to assess smoking- or BMI-related mortality which has been published earlier [11,14,30] but not the focus of the present study. The present study may have over-estimated the communicable diseases mortality which is likely to pre-exist. Thirdly, multiple biological risk factors such as hypertension, diabetes and lipid abnormalities are major predictors of cardiovascular mortality and we have no information on these variables except hypertension results published elsewhere [13]. Fourthly, the study excluded polling stations comprising upper-middle class and upper class housing complexes that were not accessible due to security issues. Similarly, the study excluded homeless persons, such as footpath dwellers, as they were generally excluded from the voter's list. Therefore, the study may not be truly representative of Mumbai or Indian population although more than 80% of the Indian population lives in social and economic circumstances as observed in the present study [31]. And finally, there are multiple measures of socioeconomic status including area-based measures, housing type, occupation, ownership, income, and others, apart from educational status. We used educational status as it has been shown to be the most robust and are the most widely used estimate [16]. Moreover, educational status is acquired in early childhood and does not change with evolving social phenotype [32] and studies in India and other low income countries have shown good correlation with multiple markers of socioeconomic status [7,27]. This is study strength. Other strengths mainly includes a population based nature of the cohort, very large sample size that is much more than many of the earlier studies, and first time use of hand-help computers (electronic diaries) for house to house data collection using face-to-face interviewers in the second most populous country in the world.

The Whitehall study reported the lowest mortality in the most educated professional and executive class and the greatest in menial workers [32-34], which was similar to what we observed for all-causes mortality in this study (Table 3, 4). This has been attributed to multiple sociological and biological determinants of health. Less literate and poor people led to unhealthy lifestyles in terms of smoking, diet and physical activity [35]. However, adjustments for several known CVD risk factors (smoking, lipids, blood pressure and diabetes) did not completely attenuate the trends and Marmot believes that the social (educational) differences in mortality could be due to factors leading to social stress such as inequality, lack of autonomy, self-esteem and social participation [36] Other social determinants of CVD health include stress, early life events, social exclusion, improper working conditions, lack of social support, addictions including tobacco and alcohol, food scarcity or excess and uneven distribution and lack of proper transport [37]. The information was not available for most of these risk factors in our study but another study from rural India reported that subjects with low educational status have inferior housing, inferior job status, improper working conditions, crowded housing and greater tobacco and alcohol use [7]. On the other hand many US studies have used educational status as a marker of socioeconomic status and reported that low educational status is an important determinant of CVD incidence and mortality. It has also been shown that those with low educational status have a lifetime risk of suffering from diseases- infections and nutrition related diseases in childhood and chronic diseases including CVD in adulthood.^16 ^Multiple socio-biological pathways have been implicated [35,38]. This is similar to the present study where both all-cause and CVD mortality was greater among the illiterate and those with low educational status. These findings are further strengthened by observation of the survival curves (Figure 2). An important observation in the present study was a clear association of illiteracy and low educational status with increased CVD mortality in men (Table 3) while the situation was not clear in women (Table 4). This could possibly be due to the fact that only a few women had education above secondary level (~6%) and the numbers of deaths observed in these groups were small. Indeed, if the data for women in more literate Groups (middle school, secondary and college) were combined in a single group the trend appears similar to those in men (Table 3, 4). Similarly, no clear associations for mortality due to IHD and stroke could also be due to lower absolute numbers. In women, the prevalence of illiteracy is high and it is known that in such circumstances, the association of literacy and chronic diseases deaths are often unclear. Previous studies in high income countries have reported that illiterate and low educational status women and men are equally at greater risk of cardiovascular deaths [39,40].

Illiteracy and low-literacy status is rampant in low-income countries [1]. Macro level evidence from high income countries suggest that improvement in literacy status, which is outside the purview of traditional public health approaches to disease prevention and management, decreases chronic diseases risk factors [41]. Greater literacy status leads to increased awareness of health risk factors at population as well as individual level. It is also associated with greater use of strategies to decrease risk factors and adherence to health promoting behaviours and therapies. This leads to decline in the three primordial as well as proximate chronic disease risk factors. Use of appropriate healthcare system and evidence based therapies for CVD treatment and control is also greater among the more literate subjects. In India and other low income countries social and biological pathways of increased CVD risk among the low educational status subjects have not been well studied and more prospective studies are needed to identify pathways to lowered risk. Over 80% of world's deaths from CVDs occur in low-and middle-income countries, such as India; where people are more exposed to risk factors leading to diseases and have less access to health care services and prevention efforts than people in high- income countries. As a result, many people die younger, often in their most productive years. In 2005, of the total projected deaths (10,362,000) in India around 28% were from CVDs. At household level, sufficient evidence is emerging to prove that CVDs and other NCDs contribute to poverty. For example, catastrophic health care expenditures for household with a family member with CVD can be 30% or more of annual household spending. Also in 2005 alone, it was estimated that India will lose $ 9 billion and will further continue to lose $ 237 billion in next 10 years in National Income from premature deaths due to heart disease, stroke and diabetes [42].

## Conclusions

Cost-effective interventions exist, and have worked in many countries: the most successful strategies have employed a range of population-wide approaches combined with interventions for individuals. Therefore, current study not only help identifying high risk group (i.e. individuals with low education) for CVDs but underscores the urgent need to direct our efforts to under privilege, which is the largest section of most developing countries like India. Additionally the study demonstrated that improving educational status may result in preventing ~9% premature male and female deaths. Amartya Sen [43], the noted economist, opines that even when an economy is poor, major health improvements can be achieved through using the available resources in socially productive way such as improving population education. Clearly improving educational status should be high priority for achieving good cardiovascular health.

## Competing interests

The authors declare that they have no competing interests.

## Authors' contributions

MSP and RG developed the concept for this paper. MSP directed the field work, was responsible for the data management, data analyses and the statistical procedures and tests, interpreted the results, conducted the literature search, and interacted with co-authors in subsequent drafts of the paper. RG was also responsible for assisting with data analysis, interpretation of results, and writing of the paper at all stages. PCG conceptualized the Mumbai cohort study, evolved field instruments and procedures, directed the field work, data management and data analyses, interpreted the results and contributed towards writing of the paper at all stages. All authors read and approved the final manuscript.

## Pre-publication history

The pre-publication history for this paper can be accessed here:

http://www.biomedcentral.com/1471-2458/11/567/prepub

## Supplementary Material

Additional file 1 Demographic details of the study subjects and comparison of subjects who were available for follow-up and those lost to follow-up Click here for file
