# Supplementary material for: Illiteracy, low educational status, and cardiovascular mortality in India
Source: BMC Public Health. 2011 Jul 15;11:567. doi: 10.1186/1471-2458-11-567 (PMC3160988; doi:10.1186/1471-2458-11-567)
Supplement: Additional file 1 — Demographic details of the study subjects and comparison of subjects who were available for follow-up and those lost to follow-up [file 1471-2458-11-567-S1.DOC]

**Additional file 1:** Demographic details of the study subjects and comparison of subjects who were available for follow-up and those lost to follow-up

| Variables | **Men (n=88658)** | | **Women (n=59515)** | |
| --- | --- | --- | --- | --- |
|  | Known vital status  (n=82660) | Lost to follow-up  (n=5998) | Known vital status  (n=58248) | Lost to follow-up  (n=1267) |
| **Age groups** |  |  |  |  |
| 35-39 | 10.0% | 2.5% | 24.8% | 27.9% |
| 40-44 | 8.4% | 2.2% | 16.9% | 16.4% |
| 45-49 | 25.8% | 39.1% | 15.3% | 16.8% |
| 50-54 | 15.8% | 21.2% | 13.3% | 12.5% |
| 55-59 | 12.2% | 11.7% | 9.9% | 8.9% |
| 60-64 | 11.0% | 11.6% | 9.2% | 8.8% |
| 65-69 | 7.7% | 6.2% | 5.1% | 3.7% |
| 70+ | 9.2% | 5.5% | 5.5% | 4.8% |
| **Educational status^*^** |  |  |  |  |
| Illiterate | 16.4% | 26.2% | 45.0% | 58.2% |
| Primary | 38.3% | 31.8% | 35.3% | 24.9% |
| Middle | 29.5% | 28.2% | 13.8% | 14.0% |
| Secondary | 9.2% | 11.4% | 4.3% | 2.3% |
| College | 6.6% | 2.4% | 1.7% | 0.6% |
| **Religion** |  |  |  |  |
| Hindu | 77.2% | 71.3% | 82.3% | 70.8% |
| Muslim | 15.6% | 22.5% | 6.4% | 20.0% |
| Buddhist | 4.2% | 4.4% | 7.7% | 5.1% |
| Christian | 2.6% | 1.7% | 3.1% | 3.9% |
| Others | 0.4% | 0.2% | 0.6% | 0.1% |
| **Mother tongue** |  |  |  |  |
| Marathi | 56.2% | 45.6% | 82.3% | 70.8% |
| Hindi | 16.1% | 27.9% | 6.4% | 20.0% |
| Gujarati | 10.1% | 3.4% | 7.7% | 5.1% |
| Urdu | 8.8% | 9.3% | 3.1% | 3.9% |
| South Indian | 8.6% | 13.8% | 0.6% | 0.1% |
| Others | 0.2% | 0.0% | 82.3% | 70.8% |
| **BMI (kg/m^2^)^**^** |  |  |  |  |
| Normal | 61.9% | 68.8% | 51.4% | 49.2% |
| Thin | 9.3% | 7.8% | 9.1% | 11.4% |
| Very thin | 4.0% | 3.3% | 4.3% | 5.8% |
| Extremely thin | 4.3% | 3.7% | 5.7% | 8.2% |
| Overweight | 17.7% | 14.7% | 22.6% | 20.4% |
| Obese | 2.7% | 1.8% | 7.0% | 5.0% |
| **Tobacco usage** |  |  |  |  |
| Never-user | 30.1% | 30.6% | 40.2% | 41.3% |
| Smokeless | 38.6% | 36.3% | 59.3% | 58.1% |
| Smoker | 16.5% | 14.9% | 0.3% | 0.3% |
| Both^***^ | 14.8% | 18.2% | 0.2% | 0.3% |

^*^ Illiterate, Primary school (≤5 years of formal education), Middle school (6-8 years), Secondary school (9-10 years) and College (>10 years)

^**^ BMI body mass index= weight (kg)/height(m)^2^; BMI (kg/m2) categories were defined as follows: extremely thin (<16.0); very thin (16.0 to <17.0); thin (17.0 to <18.5); normal (18.5 to <25.0); overweight (25.0 to <30.0); and obese (≥30.0)

^***^ includes those who smoke and use smokeless tobacco
